# The effectiveness of physical activity monitoring and distance counseling in an occupational setting – Results from a randomized controlled trial (CoAct)

**DOI:** 10.1186/1471-2458-12-344

**Published:** 2012-05-11

**Authors:** Karita Reijonsaari, Aki Vehtari, Olli-Pekka Kahilakoski, Willem van Mechelen, Timo Aro, Simo Taimela

**Affiliations:** 1 Department of Biomedical Engineering and Computational Science, Aalto University, Espoo, Finland; 2 Department of Public and Occupational Health and EMGO + Institute, VU University Medical Center, Amsterdam, The Netherlands; 3 Ilmarinen Mutual Pension Insurance Company, Helsinki, Finland; 4 Department of Public Health, Helsinki University, Helsinki, Finland; 5 Department of Industrial Engineering and Management and HEMA Institute, Aalto University, Espoo, Finland

**Keywords:** Physical activity, Health behavior, Physical activity intervention, Lifestyle intervention, Distance counseling, Work productivity, Outcomes, Randomized controlled trial

## Abstract

**Background:**

Lack of physical activity (PA) is a known risk factor for many health conditions. The workplace is a setting often used to promote activity and health. We investigated the effectiveness of an intervention on PA and productivity-related outcomes in an occupational setting.

**Methods:**

We conducted a randomized controlled trial of 12 months duration with two 1:1 allocated parallel groups of insurance company employees. Eligibility criteria included permanent employment and absence of any condition that risked the participant’s health during PA. Subjects in the intervention group monitored their daily PA with an accelerometer, set goals, had access to an online service to help them track their activity levels, and received counseling via telephone or web messages for 12 months. The control group received the results of a fitness test and an information leaflet on PA at the beginning of the study. The intervention’s aim was to increase PA, improve work productivity, and decrease sickness absence. Primary outcomes were PA (measured as MET minutes per week), work productivity (quantity and quality of work; QQ index), and sickness absence (SA) days at 12 months. Participants were assigned to groups using block randomization with a computer-generated scheme. The study was not blinded.

**Results:**

There were 544 randomized participants, of which 521 were included in the analysis (64% female, mean age 43 years). At 12 months, there was no significant difference in physical activity levels between the intervention group (n = 264) and the control group (n = 257). The adjusted mean difference was −206 MET min/week [95% Bayesian credible interval −540 to 128; negative values favor control group]. There was also no significant difference in the QQ index (−0.5 [−4.4 to 3.3]) or SA days (0.0 [−1.2 to 0.9]). Of secondary outcomes, body weight (0.5 kg [0.0 to 1.0]) and percentage of body fat (0.6% [0.2% to 1.1%]) were slightly higher in the intervention group. An exploratory subgroup analysis revealed no subgroups in which the intervention affected physical activity. No adverse events were reported.

**Conclusions:**

The intervention was not found effective, and this study does not provide support for the effectiveness of the workplace PA intervention used here.

**Trial registration:**

ClinicalTrials.gov identifier: NCT00994565

## Background

The majority of the adult population in developed countries does not meet the guidelines for physical activity recommended by American College of Sports Medicine (ACSM) [1]. A lack of physical activity has direct detrimental effects on health [2] and potential indirect effects, such as increased absenteeism from work [3-6] and loss of productivity while at work [7]. Furthermore, medically certified absence due to sickness is a strong predictor of future disability [8] and all-cause mortality [9,10].

Employers bear the financial consequences of reduced productivity and absenteeism and cover employee healthcare costs in many countries. Therefore, employers may benefit financially from implementing health intervention programs that increase the physical activity levels of their employees. This statement is supported by a review conducted for the World Economic Forum and the WHO, which estimated that interventions promoting physical activity may yield healthcare cost savings of 2.5 to 4.5 dollars for every dollar spent, and absenteeism savings of 2.5 to 4.9 dollars for every dollar spent [11]. In particular, the occupational setting has been considered useful for primary prevention and health intervention efforts, as most adults spend a major part of the day at work [12].

There is a lack of randomized controlled trials (RCTs) of lifestyle interventions in the occupational setting that evaluate productivity outcomes [11]. Non-randomized studies indicate that these interventions may reduce absence due to sickness and increase productivity at work [11]. However, selection bias may arise when allocation method other than randomization is used, meaning that the intervention and the control groups are unlikely to be comparable in non-randomized settings [13]. Previous research indicates that non-randomized studies of healthcare interventions tend to result in larger estimates of effect compared to RCTs [14]. Previous studies have concluded that when allocation is not controlled for, the results are more likely biased by baseline differences in group characteristics or confounders (e.g., motivation to change health behaviour) [15].

Inactive people are often not aware that they are insufficiently active [16]. Providing insight into their actual physical activity levels may raise awareness and could, in combination with tailored physical activity advice, stimulate a physically active lifestyle. Previous studies [17-20] have indicated that a physical activity monitor helps sedentary participants set goals and motivates them to increase their physical activity levels. However, these studies were not designed as RCTs and included mainly small populations of overweight participants or patients with type 2 diabetes. Only one RCT (n = 102 participants; 3-month intervention period) has evaluated an intervention with the same accelerometer technology that we used for activity monitoring in this study [16]. This study was conducted simultaneously with ours and we were not aware of its results while designing our research. The study by Slootmaker et al. [16] did not find a statistically significant increase in physical activity level, awareness of physical activity level, determinants of physical activity, aerobic fitness, or body composition among young and healthy office workers. Compared to Slootmaker et al. [16] our study population was substantially larger and the intervention period 9 months longer.

The RCT described here evaluated the effectiveness of a long-term 12-month intervention on levels of physical activity, sickness absence (SA), and productivity at work in a large healthy population (n = 544 participants). We expected that the intervention would increase the physical activity of the employees, reduce sickness absence, and increase productivity at work. Our study population consisted of all eligible and willing employees of a Finnish insurance company.

## Methods

### Study design and ethics

We performed a randomized, controlled trial with two parallel groups. Subjects were allocated 1:1 to each group. Both the intervention and control groups received the results of a baseline fitness test and an information leaflet on physical activity. A private company provided distance counseling regarding physical activity and provided accelerometers to monitor daily physical activity for the intervention group. The monitors were intended to be used at work and during leisure periods. Counseling was provided by two exercise specialists. The control group did not receive the distance counselling and monitoring intervention. Both groups received the results of a baseline fitness test and an information leaflet on physical activity. The groups were measured at baseline, 6 months (intervention group only), and 12 months, with the primary time point of interest being 12 months.

The objective of the intervention was to increase physical activity and consequently improve work productivity, while decreasing sickness absence. The primary outcomes measured were physical activity, work productivity, and sickness absence. The effectiveness of the intervention was assessed by comparing outcomes in the intervention and control groups at 6 months (physical activity and work productivity) and at 12 months (physical activity, productivity, and sickness absence). The Helsinki University Hospital Research Ethics Board (Coordinating Ethics Committee) approved the study, and it was performed according to the Declaration of Helsinki (2008).

### Participants

Participants were recruited from a Finnish insurance company located in Helsinki. Recruiting occurred between September 2009 and November 2009. Eligible subjects were: 1) age 18 years or older, 2) in paid employment of at least 8 h a week, 3) not scheduled to retire in the next two years and had not applied for a disability pension, 4) had completed a health risk appraisal and physical testing as a part of normal occupational healthcare, and 5) did not have the following medical conditions: pregnancy, diagnosis or treatment of cancer, or any other condition that would cause a risk to the participant’s health during testing. A complete list of the eligibility criteria is found in the study protocol [21].

Recruiting started with invitations to fill out a health risk appraisal, which was sent to all 1,116 employees of the company simultaneously. Respondents (n = 817; 73%) were invited to complete a fitness test, which was done at the workplace during normal office hours. A total of 596 subjects volunteered for fitness testing. A questionnaire concerning medical history and medication was completed before the strenuous parts of the testing. Forty-six employees were excluded for medical reasons, leaving 550 who were eligible to participate. Six subjects were further excluded from the trial: four were scheduled to retire, one was about to leave the company, and one declined to participate. Therefore, the randomized study population consisted of 544 employees.

The study design, implications of the trial, and alternative options were explained to the subjects in a cover letter. The letter emphasized that participating in the trial was voluntary and employees would get the best treatment available and full attention of the occupational health care when needed, even if they did not want to participate. It also explained that participants were free to withdraw from the trial at any point without any kind of penalty. Employees could ask questions from the research staff about the study, without any obligation to participate in the trial. Each subject who wished to participate individually signed an informed consent form. This form also allowed personal data to be collected from other data registers (health risk appraisal; physical testing; sickness absence records) so that we could add it to the research database and use it for the study. All 544 subjects signed the informed consent, but two declined the use of their sickness absence data.

### Randomization

Block randomization with blocks of ten was used. A biostatistician prepared the randomization scheme in advance by using a computer-generated randomization table. Based on the randomization scheme, two research assistants prepared sealed envelopes containing a referral to either the intervention group or the control group.

Each subject who signed the informed consent form was given a sealed envelope by the research staff according to the randomization scheme. In this way, the researchers were not able to identify group assignments. The subject opened the envelope only after receiving the fitness test results and was not allowed to change groups after randomization.

After randomization, neither the participants nor research staff were blinded to group assignments, due to the nature of the intervention. However, data entry was blinded, as sickness absence data were extracted from the company records automatically in electronic format and computer entry of self-reported data was done by a research assistant who was blinded to group assignments. Data analysts were not blinded.

### Intervention

At the beginning of the study, both groups received written results of their physical exams, and all subjects were given general information on physical activity and health. The results and informational material were briefly explained. Occupational health care continued in both groups as usual.

The intervention consisted of activity monitoring and distance counseling during the twelve-month study period. The subjects assigned to the intervention group were given a uni-axial accelerometer (PAM, model AM 200, PAM BV, the Netherlands) for monitoring daily physical activity. The PAM accelerometer has been found reliable in laboratory settings for estimating energy expenditure in treadmill walking and stair walking [22]. It produces a single index score that accumulates during the day and is continuously shown on its display. The score is a proxy measure of total daily physical activity.

At the beginning of the study, each subject set a daily PAM score goal in consultation with a counselor. The subjects installed special software on their computers which allowed them to upload their PAM scores to the service provider’s database over the Internet. Each time a subject signed on to the provider’s website, his or her PAM score goal was displayed. This goal could be modified by a user and coach throughout the intervention. On each subsequent login, the website presented all of a subject’s uploaded PAM scores and goals graphically by week or month. Subjects who did not log on to the site every two weeks to upload activity data were intended to receive a phone call or a message from the coach.

### Measurements

Both groups received a questionnaire that was used to measure physical activity and work productivity at the beginning of the study and 6 and 12 months later. A fitness test was done at baseline for both groups, at 6 months for the intervention group, and at 12 months for both groups. Sickness-related absence data was obtained from employer records. Data collection for the study started in September 2009 and continued until November 2010.

### Primary outcome measurements

The primary outcomes were 1) physical activity, 2) work productivity, and 3) sickness absence.

#### Physical activity

The volume (frequency, intensity, duration) of physical activity was assessed by a self-administered questionnaire that we created. The questionnaire enabled comparison of physical activity between the study arms. The questionnaire was based on the International Physical Activity Questionnaire (IPAQ) [23].

The volume of physical activity is often expressed as metabolic equivalents (METs). The weekly volume of physical activity (MET minutes per week) is a product of time, frequency and intensity of physical activity. MET min-per-week was calculated as follows, using the IPAQ scoring protocol: (daily minutes of walking x days per week with walking x 3.3) + (daily minutes of moderate-intensity activity x days per week with moderate intensity activity x 4.0) + (daily minutes of vigorous activity x days per week with vigorous activity x 8.0) [23]. All daily minutes exceeding 120 were truncated to 120 min, as proposed in the “Guidelines of Data Processing and Analysis of IPAQ Short Version” with the attempt to normalize skewed population data. In processing and cleaning IPAQ questionnaire answers, IPAQ guidelines were followed, except the recommendation to exclude missing data. Instead, we used multiple imputation of missing data.

#### Work productivity

Work productivity was measured with the QQ instrument [24]. Respondents assessed how much work they performed effectively during regular hours on their last regular workday as compared with usual. The quantity and quality of work productivity were measured on 10-point numerical rating scales. Two scores from 0 to 10 were given: one for quantity of work and one for quality. A score of 0 represented “nothing” and 10 on each scale represented “normal quantity/quality” [24,25]. The quantity and quality scores were multiplied with each other to obtain the QQ score, which was on a scale from 0 to 100. The QQ score correlates with objective work output [25].

#### Sickness absence

Sickness absence was operationalized as the accumulated number of sickness absence days during the study period, excluding weekends. The number of sickness absence days during the 12-month period prior to randomization was used as the baseline. This data was obtained, without medical diagnoses, from employer payroll records. Employees are required to inform the company when sick for one to three days and must provide a sickness certificate when absent for longer than three days. Data privacy was strictly followed. Records were checked for inconsistencies. Maternity/paternity leave and absence from work to care for a sick child were not counted as sickness absences.

### Secondary outcome measurements

The secondary outcomes measured were changes in body weight, waist circumference, body fat percentage, blood pressure, and aerobic fitness. These variables were measured during the fitness test at baseline, at 12 months for both groups, and at 6 months for the intervention group. Details on these measures have been described in the study protocol [21].

### Sample size calculations

The sample size calculation was based on the following predefined assumptions [21]. The standard deviation for the IPAQ score in our population was estimated to be 1500 MET min-per-week. We considered a difference of 400 MET min-per-week between the intervention and control groups to be practically significant, detectable with 85% power in two-tailed tests with the alpha of 0.05 for a sample of 253 employees in each group; the standardized effect size is 0.27. Therefore, the obtained study population of 544 subjects was adequate for detecting a practically significant difference with a 7% drop-out rate.

### Statistical analyses

The intervention effect was estimated based on the intention-to-treat principle. Subjects who left for maternity leave, resigned, or retired by the end of the study period were excluded from analysis. The two subjects who declined the use of their sickness absence data were excluded from analysis of sickness absence.

A high number of fitness tests and questionnaires were missing at the 12-month time point. We assumed missingness-at-random and did multiple imputation with Gaussian expectation-maximization algorithm using MATLAB toolbox *pmtk3*[26]. The number of random imputations used was 20. Imputation covariates included items from the previous tests and questionnaires, such as age, gender, body-mass index, and maximal oxygen uptake. Negative values from imputation were truncated to zero. Imputed values of work productivity were truncated to the allowed range (0–100). We did sensitivity analysis with regard to the imputation procedure by doing a complete case analysis, that is, using data only from subjects who had completed the trial and had no missing data.

For physical activity, work productivity, and each secondary outcome, the difference between the intervention and control groups was estimated using ANCOVA, adjusting for baseline. The analyses were done using the statistical software R [27]. As the number of observations was relatively large compared to the number of covariates, the results could be interpreted as approximately Bayesian with non-informative priors for the parameters. For sickness absence (SA), we used the hurdle negative binomial model to account for its discrete and non-Gaussian distribution. Due to the complex distribution, full Bayesian inference with hierarchical prior was used.

Hurdle models assume a two-stage process [28]. In our analysis, the first process (the zero process) determined if a person has any SAs. The second process (the count process) determined the number of non-zero SA days. We used logistic regression and zero truncated negative binomial regression to model the zero and count processes, respectively. In contrast to Poisson regression, negative binomial regression allows overdispersion, which is common in count data. The SA days of the previous year were adjusted for by including them as a covariate in the model. We also included a random effect component to model person-specific levels for SA days. The hurdle negative binomial model was implemented using MATLAB’s *GPstuff* toolbox [29].

We also performed an exploratory subgroup analysis to detect possible effect modifiers and mediators. The effect modifiers were personal characteristics (age and gender), self-rated level of physical activity, job characteristics (specialist/manager), and sick leave days in the past year, each assessed at baseline. We used physical activity at 12 months as the outcome of this analysis.

Finally, we assessed whether adherence to the intervention was a mediator for the effect on sickness absences. The study population was divided into adhering and non-adhering groups. Those in the adhering group returned the questionnaire and had a physical exam at 12 months. We used the number of sickness absence days during the follow-up year as the outcome, as this information was also available for the non-adhering group. We then assessed the interaction adherence x group assignment using a hurdle negative binomial model.

For differences between the groups, we report the baseline-adjusted mean difference and its 95% Bayesian credible interval (CI). 95% CI is such interval that the difference is within the interval with 95% probability.

## Results

**Figure 1:**
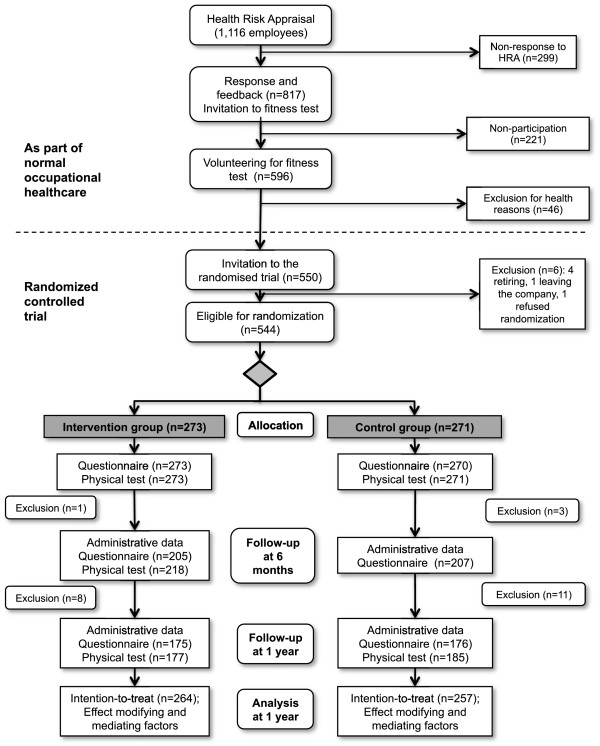
Participant flow.

The randomized study population consisted of 544 subjects. A total of 273 subjects were randomized into the intervention group, and 271 subjects were in the control group. During the 12-month trial, 23 employees retired, resigned, or left for maternity leave. They were removed from the intention-to-treat analysis, leaving 521 subjects: 264 were in the intervention group and 257 were in the control group. The participant flow is presented in Figure 1.

At baseline, the average age of participants was 43 years (range 23–64 years). 64% of subjects were female, and 90% were clerical employees. Eighteen percent were physically inactive [30] and 13% were smokers. The average body mass index (BMI) was 25 (standard deviation: 4), and 70% of the subjects met the ACSM guideline recommendations for physical activity sufficient to promote and maintain health. This recommendation is a minimum of 600 MET minutes/week of at least moderate intensity exercise [1]. We found no relevant differences between the intervention and the control groups at baseline (Table 1).

**Table 1:** Baseline characteristics of intervention and control groups: mean (standard deviation) or count (percentage) within group

	**Baseline**	
	**Control**	**Intervention**
n	257	264
Age (years)	44 (10)	43 (10)
Gender (female)	154 (60%)	180 (68%)
Clerical employees	231 (90%)	236 (89%)
Body-mass index (kg/m^2^)	25 (4)	25 (4)
ACSM guideline	185 (72%)	182 (69%)

### Adherence

The loss to follow-up was considerable in both groups (Table 2). At 12 months, 362 subjects (69%) underwent physical testing and 351 subjects (68%) returned the questionnaire measuring physical activity and work productivity.

**Table 2:** Loss to follow-up at baseline, 6 months, and 12 months in the intervention and the control groups

		**Baseline**	**6 months**	**12 months**
Questionnaire	Intervention	264 (100%)	201 (76%)	175 (66%)
	Control	257 (100%)	200 (78%)	176 (68%)
Physical test	Intervention	264 (100%)	215 (81%)	177 (67%)
	Control	257 (100%)		185 (72%)

The use of the web-based service decreased with time. Subjects in the intervention group averaged 15 logins during the last 6 months of the trial (0.6 times per week). Entries of physical activity were manually added to the database for 14% of the days in the first six months and 9% of the days in the second six-month period. The amount of communication between the coaches and the participants also decreased. Coaches sent an average of 7.2 and 6.1 personal messages to each subject during the first and the second six months, respectively. Likewise, the subjects averaged 4.3 and 1.7 personal messages to the coaches during the first and the second six months.

### Effectiveness of the intervention

#### Primary outcomes

The results at baseline and 12 month follow-up are presented in Table 3. The intervention was not found effective on physical activity. The adjusted mean difference between the intervention and control groups at 6 months was −365 MET min/week (95% CI: -733 to 3; negative values favor control group) and at 12 months −207 MET min/week (−531 to 116). We found no productivity difference between the intervention and control groups, either. The adjusted mean differences in the QQ index were 1.3 (−2.0 to 4.7) and −1.1 (−4.9 to 2.8) at 6 months and 12 months, respectively.

**Table 3:** Primary outcomes in intervention and control groups: mean (standard deviation) or count (percentage) within group and adjusted differences between groups at 6 and 12 months

	**Baseline**		**6 months**		**12 months**		**Adjusted difference (95% CI)**	
	**Control**	**Intervention**	**Control**	**Intervention**	**Control**	**Intervention**	**6 months**	**12 months**
n	257	264	257	264	257	264		
Physical activity	2,258	2,083	2,440	1,995	2,338	2,047	−365	−207
(IPAQ, MET min/week)	(1,484)	(1,439)	(1,966)	(1,673)	(1,762)	(1,650)	(−733 to 3)	(−531 to 116)
Work productivity	80	81	83	85	81	81	1.3	−1.1
(QQ index, range 0–100)	(22)	(20)	(20)	(18)	(20)	(20)	(−2.0 to 4.7)	(−4.9 to 2.8)
Sickness absence								
None (%)	30	25			24	28		
Mean (days)	7.4	6.5			9.7	6.9		0.0 (−1.2 to 0.9)
Upper quartile (days)	8	8			10	8		
Maximum (days)	200	88			219	87		

Negative differences favor control group.

The adjusted mean difference in accumulated sickness absence days during the 12 months between the intervention and control groups was 0.0 days (−1.2 to 0.9).

#### Secondary outcomes

Table 4 presents the secondary outcomes. Body weight and fat percentage increased slightly in the intervention group. The adjusted mean differences between intervention and control groups were −0.5 kg (95% CI: −1.0 to 0.0; negative values favor control group) and −0.6% (−1.0% to −0.2%). No significant differences between the groups in other secondary outcome measures were found. The adjusted mean differences were 0.0 ml/kg/min (−0.6 to 0.6) for maximal oxygen uptake, −0.1 cm (−0.7 to 0.6) for waist circumference, 0.3 mmHg (−1.8 to 2.4) for systolic blood pressure, and 0.7 mmHg (−0.5 to 2.0) for diastolic blood pressure.

**Table 4:** Secondary outcomes in the intervention and the control group: means and standard deviations (in brackets) within group and adjusted differences between groups at 12 months

	**Baseline**				**12 months**				**Adjusted difference (95% CI)**
	**Control**		**Intervention**		**Control**		**Intervention**		**12 months**
n	257		264		257		264		
Maximal oxygen uptake (ml/kg/min)	39 (8)		38 (8)		40 (9)		39 (8)		0.0 (−0.6 to 0.6)
Body weight (kg)	73 (13)		71 (14)		73 (13)		72 (13)		−0.5 (−1.0 to 0.0)
Waist circumference (cm)	86 (11)		85 (11)		86 (10)		85 (10)		−0.1 (−0.7 to 0.6)
% body fat	27 (9)		27 (9)		27 (8)		28 (8)		−0.6 (−1.0 to −0.2)
Systolic blood pressure (mm Hg)	138 (14)		136 (14)		137 (14)		135 (14)		0.3 (−1.8 to 2.4)
Diastolic blood pressure (mm Hg)	83 (9)		82 (9)		82 (8)		80 (9)		0.7 (−0.5 to 2.0)

Negative differences favor control group.

### Exploratory subgroup analysis

**Figure 2:**
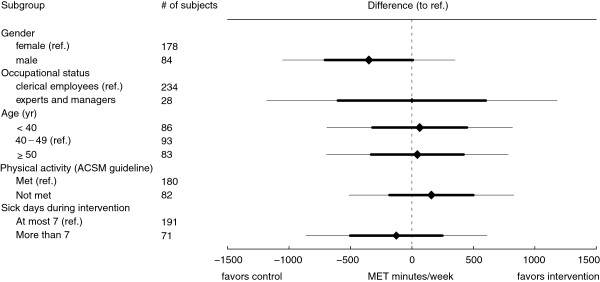
**Results of the subgroup analysis.** The subgroups were compared to a reference level of a 40–49 year old female in control group meeting the ACSM guideline recommendations for physical activity, and has at most seven sick days during the period. Thick lines show the 68% confidence intervals for the difference, and thin lines show the 95% confidence intervals. The diamond area reflects the subgroup size. Physical activity was measured as metabolic equivalents (METs) per week.

The effect was not modified by gender, job characteristics, age, self-rated level of baseline physical activity, or sickness absence days in the past year (Figure 2).

Adherence to the intervention did not mediate sickness absence: the mean difference between the adhering and non-adhering subgroups was 0.0 days (95% CI: -1.2 to 0.9; negative values favor control group).

### Sensitivity analyses

Sensitivity of the results was assessed by doing the analyses both with multiply imputed data and complete cases. The results did not differ.

### Adverse events

No adverse events were reported during the interventions.

## Discussion

### Main findings

Daily monitoring of physical activity and distance counseling as a lifestyle intervention was not found effective on physical activity, work productivity, or sickness absence. At 12 months, there was no difference in physical activity levels between the intervention group and the control group. Furthermore, exploratory subgroup analysis did not reveal any effects, either. Adherence to the intervention was not found to mediate the effect on sickness absence. The secondary outcomes body weight and fat percentage increased slightly in the intervention group, contrary to our expectations.

A majority of the subjects were physically active when the study started: 70% met the ACSM guidelines for physical activity at baseline. This fact could partly explain the absence of intervention effects. However, subgroup analysis did not show that employees with low physical activity level at baseline were more likely to increase their physical activity than the employees who were already active.

An explanation for the intervention’s limited effectiveness may be that it was not intense enough to establish significant changes in physical activity and maintain them. The technology used for remote monitoring of physical activity, without human contact, may not have been engaging enough to change daily habits.

### Strengths and weaknesses of the study

The main strength of the present study was the fact that we were able to implement it in a real workplace; closely resembling the way the commercial intervention would have been implemented had there not been research involved. Another strength of this study was that all eligible employees in the target cohort were offered the opportunity to participate. The participation rate (49%) was similar to rates in other health promotion studies in occupational populations [31].

One limitation of the study was that most of the subjects were relatively active and healthy when the study began. This is not surprising; physical activity interventions tend to attract people who are young, healthy, and already sufficiently physically active to maintain their health [16,32-34]. Our participants were, on average, younger and slimmer than non-participants, fewer of them were inactive, and they smoked less and reported fewer health problems at the outset than non-participants (data not shown). We also excluded 46 employees from the baseline fitness test due to health reasons. Thus, our results may have been affected by selection bias, both from self-selection and exclusion of certain potential participants. These possibilities may limit the generalizability of our findings.

Another limitation of this study is that we did not collect information about the desire of participants to become more active. We also did not determine how participants felt about the physical activity monitoring and distance counseling. A previous study [16] using the same monitoring technology found that a large part of the intervention group did not find the advice appealing. Our analysis might have benefited from additional qualitative measures on individual motivations to participate or not to participate, experiences with the service and feedback on the quality of the advice given. Qualitative approach, such as interviewing the subjects, may have also been able to explain some of the findings favouring the control group.

Our control group was not truly a non-intervention group because the subjects received the results of a baseline physical test and an information leaflet on physical activity. However, we believe that lasting changes in physical activity are unlikely to occur just because some information was provided once.

Although validated instruments were used to measure physical activity and work productivity [23,24], their sensitivity may be suboptimal [35,36]. Also, relying on self-reported measures may add uncertainty to results, as self-reported measures are prone to recall and social desirability biases. However, this problem would apply to both intervention arms. On the other hand, the sickness absence data were accurate and consistent, as they were obtained from company records.

Our study had a longer intervention period than many previous studies [37], allowing us to assess the long-term engagement of the subjects with the intervention. The website login frequency (0.6 times per week during the last 6 months of the trial) was lower than in many shorter term studies (range 0.7 to 1.5 times per week) [16,32,38]. Unfortunately, we were not able to assess the login frequency during the first 6 months of the trial due to technical problems.

Although messaging activity and the number of logins declined throughout the study period, a reasonable adherence was maintained. At 12 months, 69% of the study population underwent physical testing and 67% returned the questionnaire measuring physical activity and work productivity.

## Conclusions

The intervention studied here consisted of daily activity monitoring and distance counseling. The intervention is commercially available and widely used in the occupational setting, and the service providers have advertized its efficacy. Therefore, the empirical basis of our study questions was to examine the efficacy of the intervention in a real-life setting where the service is currently used.

The intervention was not found effective on physical activity, work productivity, or sickness absence among the employees of a Finnish insurance company. Further study is required to determine the precise reasons for this result. They may include selective participation, motivational measures, insufficient adherence to the program, or the quality and appropriateness of the activity monitoring or tailored advice. Our current results do not justify a wider implementation of this intervention among healthy and physically active office workers.

## Competing interests

KR, AV, O-PK and TA declare that they have no competing interests. ST is the founder and CEO in Evalua International Ltd. that was responsible for the health risk appraisal used. ST and WvM are directors of Evalua Nederland b.v.

## Authors’ contributions

ST is the principal investigator. KR developed the idea for the study and obtained funding for research. KR and ST designed the conduct of the study and authored the article. O-PK performed the statistical analyses and co-authored the corresponding parts of the article, and AV advised on the analyses. WvM and TA provided expert comments. All authors have reviewed and commented the paper during the writing process. All authors have also read and approved the final manuscript.

## Pre-publication history

The pre-publication history for this paper can be accessed here:

http://www.biomedcentral.com/1471-2458/12/344/prepub
